# MVSO-PPIS: a structured objective learning model for protein–protein interaction sites prediction via multi-view graph information integration

**DOI:** 10.1093/bioinformatics/btaf470

**Published:** 2025-09-01

**Authors:** Shuang Wang, Tianle Ma, Kaiyu Dong, Peifu Han, Xue Li, Junteng Ma, Mao Li, Tao Song

**Affiliations:** Qingdao Institute of Software, College of Computer Science and Technology, China University of Petroleum (East China), Shandong 266580, China; Shandong Key Laboratory of Intelligent Oil & Gas Industrial Software, Shandong 266580, China; Qingdao Institute of Software, College of Computer Science and Technology, China University of Petroleum (East China), Shandong 266580, China; Qingdao Institute of Software, College of Computer Science and Technology, China University of Petroleum (East China), Shandong 266580, China; Qingdao Institute of Software, College of Computer Science and Technology, China University of Petroleum (East China), Shandong 266580, China; Qingdao Institute of Software, College of Computer Science and Technology, China University of Petroleum (East China), Shandong 266580, China; Qingdao Institute of Software, College of Computer Science and Technology, China University of Petroleum (East China), Shandong 266580, China; Qingdao Institute of Software, College of Computer Science and Technology, China University of Petroleum (East China), Shandong 266580, China; Qingdao Institute of Software, College of Computer Science and Technology, China University of Petroleum (East China), Shandong 266580, China; Shandong Key Laboratory of Intelligent Oil & Gas Industrial Software, Shandong 266580, China

## Abstract

**Motivation:**

Predicting protein–protein interaction (PPI) sites is essential for advancing our understanding of protein interactions, as accurate predictions can significantly reduce experimental costs and time. While considerable progress has been made in identifying binding sites at the level of individual amino acid residues, the prediction accuracy for residue subsequences at transitional boundaries—such as those represented by patterns like singular structures (mutation characteristics of contiguous interacting-residue segments) or edge structures (boundary transitions between interacting/non-interacting residue segments) still requires improvement.

**Results:**

we propose a novel PPI site prediction method named MVSO-PPIS. This method integrates two complementary feature extraction modules, a subgraph-based module and an enhanced graph attention module. The extracted features are fused using an attention-based fusion mechanism, producing a composite representation that captures both local protein substructures and global contextual dependencies. MVSO-PPIS is trained to jointly optimize three objectives: overall PPI site prediction accuracy, edge structural consistency, and recognition of unique structural patterns in PPI site sequences. Experimental results on benchmark datasets demonstrate that MVSO-PPIS outperforms existing baseline models in both accuracy and structural interpretability.

**Availability and implementation:**

The datasets, source codes, and models of MVSO-PPIS are all available at https://github.com/Edwardblue282/MVSO-PPIS.

## 1 Introduction

Notably, drug design has become an important branch of bioinformatics, with an increasing body of research focusing on integrating computational approaches into this field, such as the work by Wang *et al.* (2024, 2025), Lai *et al.* (2025), and Tang *et al.* (2024). Identifying protein–protein interaction sites (PPIS) is crucial for understanding protein functions, interaction networks, disease mechanisms, and drug design. While traditional experimental methods such as X-ray crystallography and yeast two-hybrid screening are effective, they are time-consuming and expensive. Thus, developing rapid and accurate computational methods for PPIS prediction is essential for assisting experimental techniques, improving protein docking, predicting functions, and aiding drug development (Li *et al.* 2020, O’Connor *et al.* 2023).

Deep learning-based methods for protein–protein interaction site (PPIS) prediction can be broadly classified into sequence-based and structure-based approaches. Sequence-based methods primarily rely on the modeling of primary or secondary protein structures. Representative techniques include convolutional neural networks (CNNs) (Li *et al.* 2021), recurrent neural networks (RNNs) (Zhang *et al.* 2019, Li *et al.* 2021, Hou *et al.* 2023, Kang *et al.* 2023), and other sequential models (Khan *et al.* 2022, Hosseini *et al.* 2024). To address the limitations of CNNs in modeling long-range dependencies, RNN-based models have been introduced. Li *et al.* (2021) combines CNN and RNN architectures to capture both local and global features, while Zhang *et al.* (2019) utilizes a simplified long short-term memory (LSTM) network to improve performance on imbalanced datasets. Nevertheless, the reliance of these methods on sequence-derived features has led to performance saturation, motivating a paradigm shift toward the incorporation of tertiary structural information (Zhang *et al.* 2011).

Structure-based methods exploit 3D protein structural data to provide a more comprehensive representation of residue environments. For example, Yuan *et al.* (2021) formulates the PPIS prediction task as a node classification problem on residue-level graphs using graph convolutional networks (GCNs). Zhou *et al.* (2023) extends the graph attention network (GAT) framework by integrating edge features, thereby enhancing its ability to model spatial relationships and improve invariance. Similarly, Zeng *et al.* (2024) incorporates GraphHeat diffusion and Generalized PageRank (GPR) techniques to enhance predictive performance and generalization capability. These structure-based approaches (Zhang *et al.* 2011, Yuan *et al.* 2021, Wang *et al.* 2022, Zhou *et al.* 2023, Han *et al.* 2024, Zeng *et al.* 2024) leverage geometric and topological information, demonstrating superior accuracy compared to their sequence-based counterparts. Despite substantial progress, significant challenges persist in the precise identification of local subsequences—namely, singular structures (e.g. “010” or “101”), which encode mutation-induced transitions within contiguous interacting-residue segments, and edge structures (e.g. “01” or “10”), which demarcate the boundaries between interacting and non-interacting residue segments. In this binary notation, 1 represents an interacting residue, and 0 represents a non-interacting residue.

To address the challenge of existing research that neglects the structural aspects of protein-protein interaction site (PPIS) predictions and to fully utilize the hidden information within protein graphs for more accurate PPIS identification, we propose an innovative prediction method named MVSO-PPIS. Unlike AGAT-PPIS and GHGPR-PPIS, which relies solely on the original protein view, MVSO-PPIS adopts a dual-view strategy: a subgraph view feature extraction module and an enhanced graph attention feature extraction module based on the original protein graph. This muti-view approach not only captures the fine-grained features of protein binding sites but also enhances the correlation between the binding site regions and original protein graph.

Besides, the model introduces a unique Attention fusion strategy to generate a comprehensive protein feature representation that encompasses protein substructure information and enhanced graph attention information, which could provide richer and more structured information for PPIS prediction tasks. In order to effectively identify edge protein sites within protein sequences, a joint three-part structural learning objective is proposed, enabling MVSO-PPIS to produce more accurate and structurally coherent PPIS prediction results. In contrast, AGAT-PPIS and GHGPR-PPIS rely solely on a cross-entropy-based learning strategy, limiting their ability to capture complex structural dependencies.

## 2 Materials and methods

### 2.1 Datasets

In this study, the datasets used were assembled from established protein-protein complexes within the Protein Data Bank (PDB), aligning with the foundational dataset utilized in the antecedent research (Yuan *et al.* 2021, Zhou *et al.* 2023). The dataset comprises a training set (Train_335-1) and several test sets (Test_60, Test_315, and Ubtest_31-6). In our work, the Train_335-1 dataset is predominantly used, while the other datasets are reserved for assessing model performance, with Test_60 serving as the main benchmark test set and the remaining datasets used to examine the model’s generalization capability. In the model comparisons, Btest_31-6 is constructed based on the monomeric structures of 25 proteins from Test_60, which correspond to those in UBtest_31-6. Specifically, out of the 60 proteins in Test_60, 31 have known monomeric structures in PDB, forming an additional unbound test set (UBtest_31). We additionally utilize the PP-1001_Train and PP-250_Test datasets, both derived from the Protein–Protein Docking Benchmark 5.5 (Vreven *et al.* 2015) and Dockground databases (Kundrotas *et al.* 2018). The PP-1001_Train dataset is primarily used for model training, while the PP-250_Test dataset is reserved for evaluating the model’s performance with ProtT5 embeddings. Detailed information regarding the datasets can be found in Table 1, available as supplementary data at *Bioinformatics* online.

### 2.2 Protein representation

Each protein is represented as an undirected graph G(X,E,A). In this graph, the nodes *X* represent the set of amino acids of the protein, while the edges *E* indicate the connections between two amino acids. The adjacency matrix *A* signifies whether there is a connection between two amino acids in the protein.

Node features are derived by integrating sequence and structural information, as in the AGAT-PPIS method (Zhou *et al.* 2023). Sequence features include the Position-Specific Scoring Matrix (PSSM), generated using PSI-BLAST (Altschul 2012) against the UniRef90 database, and the Hidden Markov Model (HMM) matrix, constructed with HHblits v3.0.3 (Remmert *et al.* 2011) using the UniClust30 database to capture amino acid insertions and deletions.

Structural features are encoded through DSSP, atomic properties (AF), and Pseudo-Position Embedding Features (PEF). DSSP features are derived from the protein’s 3D structure, producing a 14D vector with secondary structure, solvent accessibility, and backbone dihedral angles. Atomic features describe residue atoms (excluding hydrogens), including atomic mass, B-factor, side-chain participation, electronic charge, and van der Waals radius. Pseudo-Position Embedding Features are based on the side-chain centroid (SC) coordinates, derived from the PDB, forming a N×3 matrix of residue positions.

These sequence and structural features combine to form a node feature matrix *X* of size L×62, where *L* is the number of amino acids in the protein, and the feature dimensionality is 62.

Edge features describe spatial relationships between amino acid nodes. Using PDB-derived coordinates, Euclidean distances between residues are computed, with edges established if the distance is below a cutoff [14 Å, as per (Yuan *et al.* 2021)]. This generates an adjacency matrix *A*, with 1 indicating a connection and 0 indicating no edge. The specific steps for constructing edge features are detailed in the Supplementary Material.

### 2.3 The architecture of MVSO-PPIS

**Figure 1.**
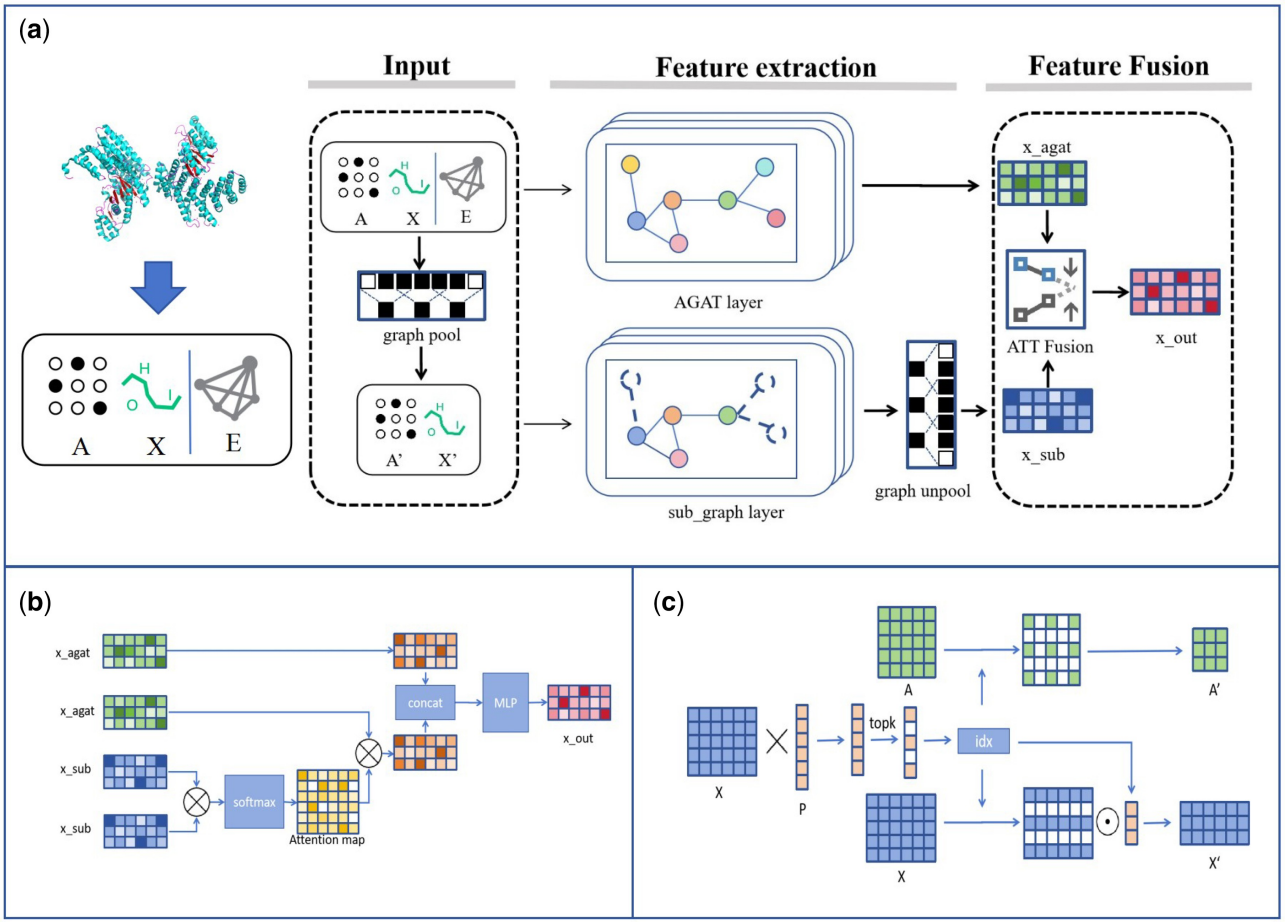
The architecture of MVSO-PPIS. (a) The overall architecture of MVSO-PPIS, with A being the adjacency matrix of the protein graph, X representing the feature matrix of the protein graph nodes, and E denoting the edge feature matrix of the protein graph. (b) The structure of the ATT Fusion module. (c) The processing procedure of the graph pool module.

The overall framework of MVSO-PPIS, illustrated in Fig. 1a, consists of three main components. The first module is the multi-view feature extraction, comprising two channels: AGAT for global graph information and GCN for local subgraph features. The second component is the multi-view feature fusion module, where outputs from both channels(reflecting sequence and structural information) are integrated via the ATT Fusion module. This fusion enhances feature completeness by retaining subgraph focus while incorporating non-subgraph node information. The third component is the MLP classifier, which uses the fused features to generate the final prediction. An overview of the protein graph subgraph construction is illustrated in Fig. 1a, and the detailed procedures are provided in the Supplementary Material.

### 2.4 Protein subgraph feature extraction with convolutional module

In order to integrate structural information between subgraph nodes, the SubGraph Convolutional Module is used. The node feature matrix $X^{\prime }$ and adjacency matrix $A^{\prime }$ of the protein subgraph are obtained after graph pooling, and they are input into the SubGraph Convolutional Module, and the output is obtained with features containing subgraph information, denoted as $H^{\left(l\right)}$. Referring to Chen *et al.* (2020), the computation mechanism of the SubGraph Convolutional Module is specifically represented by Equation (1). Equation (1) is jointly determined by $H_{\mathrm{sub}}^{\left(l\right)}$, $H_{\mathrm{sub}}^{\left(0\right)}$ and *P*. The update of $H_{\mathrm{sub}}^{\left(l\right)}$ is influenced by the adjacency matrix $A^{\prime }$, which allows the module to incorporate structural information of the subgraph during learning.


(1) $$H_{\mathrm{sub}}^{\left(l+1\right)}=\sigma ((1-\alpha )PH_{\mathrm{sub}}^{\left(l\right)}+\alpha H_{\mathrm{sub}}^{\left(0\right)})((1-\beta_{l})I_{n}+\beta_{l}W^{\left(l\right)}))$$

where *D* is the degree matrix of $A^{\prime }$; $H_{\mathrm{sub}}^{\left(0\right)}$ represents the node feature matrix $X^{\prime }$; $I_{n}$ is the identity matrix. $P=D^{-1/2}A^{\prime }D^{-1/2}$. α and $\beta_{l}$ are hyperparameters, with $\beta_{l}=\text{log}(\frac{\lambda }{l}+1)$ being determined by another hyperparameter λ.

### 2.5 Protein original graph feature extraction with AGAT module

In order to fully integrate the overall structural information of proteins, the augmented graph attention network (AGAT) is utilized. The AGAT module, as referenced in AGAT-PPIS (Zhou *et al.* 2023), is composed of two parts: an enhanced graph attention layer and a graph convolutional layer with initial residual and identity mapping. The enhanced graph attention layer incorporating edge information into the attention calculation formula to enhance the network training based on the graph attention network (GAT) (Veličković *et al.* 2018). The graph convolutional layer with initial residual and identity mapping is an extension of GCNII (Chen *et al.* 2020). The specific calculation steps are referenced in Equation (2), which introduces richer initial node embedding information at each layer of the model, thereby further avoiding over-smoothing.


(2) $$\begin{array}{c} H_{\mathrm{AGAT}}^{\left(l+1\right)}=\sigma ((1-\beta_{l})((1-\alpha )H_{\mathrm{AGAT}}^{\left(l\right)}+\alpha H_{\mathrm{AGAT}}^{\left(0\right)})\\ \;+(\beta_{l}(H_{\mathrm{AGAT}}^{\left(l\right)}||H_{\mathrm{AGAT}}^{\left(0\right)})W^{\left(l\right)})) \end{array}$$

where $H_{\mathrm{AGAT}}^{\left(l+1\right)}$ and $H_{\mathrm{AGAT}}^{\left(l\right)}$ represent the node features of the (l + 1)-th and l-th layers, respectively; $W^{\left(l\right)}$ represents the learnable parameters; σ is defined as the ReLU activation function; α and $\beta_{l}$ are hyperparameters, with $\beta_{l}=\text{log}(\frac{\lambda }{l}+1)$ being determined by another hyperparameter λ.

### 2.6 ATT fusion module for multi-view feature fusing

While the SubGraph Convolutional Module captures key point sets within subgraphs, its extracted features lack completeness. To mitigate this, we propose an attention-based fusion strategy (Fig. 1b), integrating subgraph features with full-graph representations. Specifically, subgraph features $H_{\mathrm{sub}}$ serve as attention weights to dynamically fuse with complete graph features $H_{\mathrm{AGAT}}$. This preserves critical subgraph information while compensating for missing context, enhancing feature robustness. For computational details, see Equation (3), where $d_{\mathrm{sub}}$ denotes $H_{\mathrm{sub}}$’s feature dimension.


(3) $$\begin{array}{c} H_{\text{ATT}}=\text{Attention}(H_{\mathrm{sub}},H_{\mathrm{sub}},H_{\mathrm{AGAT}})\\ =\text{softmax}\left(\frac{H_{\mathrm{sub}}H_{\mathrm{sub}}^{T}}{\sqrt{d_{\text{sub}}}}\right)H_{\mathrm{AGAT}} \end{array}$$

The final output of the model is obtained by concatenating $H_{\mathrm{ATT}}$ and $H_{\mathrm{AGAT}}$, and then inputting the result into a three-layer multilayer perceptron (MLP). The specific calculation formula is:


(4) $$H_{\text{out}}=\text{mlp}(H_{\text{ATT}}||H_{\mathrm{AGAT}})$$

### 2.7 The structured objective learning of MVSO-PPIS

Although deep learning-based methods have achieved high residue-level accuracy in protein binding site prediction, their biological applicability warrants further scrutiny. Effective predictions should ensure both precise residue annotations and structural coherence. In particular, maintaining the physical plausibility and functional consistency of the 3D structure is essential for reliable functional interpretation.

The key challenge lies in accurately modeling boundary transitions between binding and non-binding residues. These transitions exhibit specific spatial patterns – “01”/“10” transitions represent boundary edges between interacting and non-interacting regions, while “010” patterns correspond to isolated binding residues that often indicate structurally sensitive points. Traditional approaches typically treat residue prediction as independent classification tasks, which fails to capture these biologically meaningful patterns and often leads to fragmented, physically implausible predictions.

**Figure 2.**
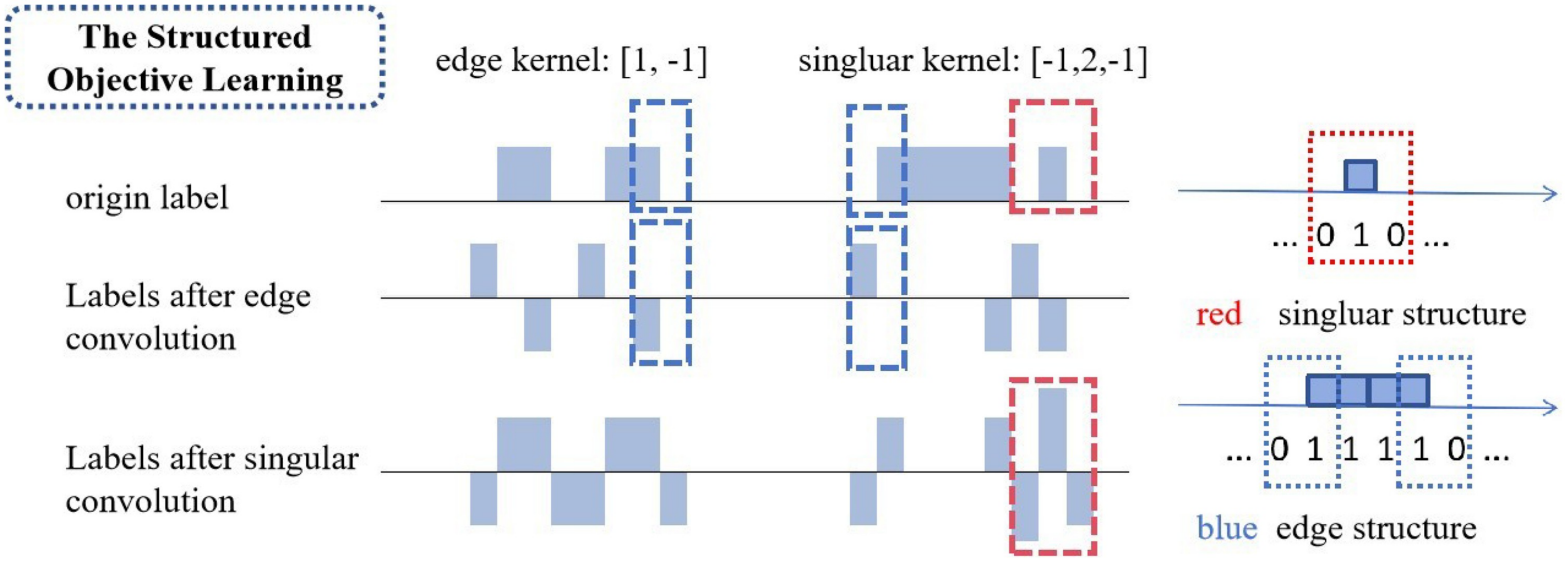
The example of edge convolution and singular convolution. In the figure, 1 represents interacting residues, while 0 represents non-interacting residues.

Our structured learning framework addresses these limitations through three synergistic mechanisms. First, pattern-specific feature learning uses specialized convolutional kernels ($K_{\mathrm{edge}}^{i}$ and $K_{\mathrm{singular}}^{i}$) to explicitly detect biologically meaningful transition patterns at multiple scales, analogous to edge and blob detection in computer vision (Fig. 2). These kernels directly encode prior knowledge about boundary geometries in protein interfaces. Second, hierarchical loss optimization through our composite loss function [Equation (5)] simultaneously enforces both local accuracy and global structural consistency: the correctness objective ($L_{\mathrm{correctness}}$) maintains residue-level prediction accuracy, while the edge structure objective ($L_{\mathrm{edge}}$) preserves boundary continuity through “01”/“10” pattern matching, and the singular point objective ($L_{\mathrm{singular}}$) ensures proper spatial distribution of isolated binding sites (“010” patterns). Third, the framework incorporates biophysical constraints by comparing predicted and true structural patterns at multiple scales through convolutional operations [Equations (6), (7), and 8], promoting physically plausible configurations.


(5) $$L_{\mathrm{total}}=L_{\mathrm{correctness}}+\alpha *(0.5*L_{\mathrm{edge}}+0.5*L_{\mathrm{singular}})$$

(6) $$L_{\text{correctness}}=-\frac{1}{n}\sum_{i=1}^{n}\sum_{c=1}^{m}t_{i,c}\;\text{log}(y_{i,c})$$

(7) $$L_{\mathrm{edge}}=\sum_{i=1}^{3}MSE(\mathrm{conv}(k_{\mathrm{edge}}^{i},H_{\text{out}}),\mathrm{conv}(k_{\mathrm{edge}}^{i},\;T))$$

(8) $$L_{\text{singular}}=\sum_{i=1}^{3}MSE(\mathrm{conv}(k_{\text{singular}}^{i},H_{\text{out}}),\mathrm{conv}(k_{\text{singular}}^{i},T))$$

## 3 Results

### 3.1 Comparison of model performance with other models

On the independent dataset Test_60, the performance of MVSO-PPIS was compared with other protein-protein interaction (PPI) site predictors. In order to comprehensively and objectively evaluate the performance of the model, some comparative experiments were conducted. The methods considered include sequence-based models: PSIVER (Murakami and Mizuguchi 2010), ProNA2-020 (Qiu *et al.* 2020), SCRIBER (Zhang and Kurgan 2019), DLPred (Zhang *et al.* 2019), Seq-InSite (Hosseini *et al.* 2024) and DELPHI (Li *et al.* 2021); and structure-based models: DeepPPISP (Zeng *et al.* 2020), SPPIDER (Porollo and Meller 2007), MaSIF-site (Gainza *et al.* 2020), Graph-PPIS (Yuan *et al.* 2021), RGN (Wang *et al.* 2022), DeepProSite (Fang *et al.* 2023), AGAT-PPIS (Zhou *et al.* 2023), and GHGPR-PPIS (Zeng *et al.* 2024). According to Table 1, seven evaluation metrics were used to assess the performance of MVSO-PPIS on the benchmark dataset. The experimental results indicate that MVSO-PPIS outperformed the AGAT-PPIS method by 1.2% in Accuracy (ACC), 3.5% in Precision, 2.7% in Recall, 3.2% in F1 score, 3.9% in Matthews Correlation Coefficient (MCC), 0.6% in Area Under the Receiver Operating Characteristic Curve (AUROC), and 0.9% in Area Under the Precision-Recall Curve (AUPRC).

**Table 1.** Performance comparison with other models on Test_60.

Method	ACC	Precision	Recall	F1	MCC	AUROC	AUPRC
PSIVER	0.561	0.188	0.534	0.278	0.074	0.573	0.190
ProNA2020	0.738	0.275	0.402	0.326	0.176	N/A	N/A
SCRIBER	0.667	0.253	0.568	0.350	0.193	0.665	0.278
DLPred	0.682	0.264	0.565	0.360	0.208	0.677	0.294
DELPHI	0.697	0.276	0.568	0.372	0.225	0.699	0.319
DeepPPISP	0.657	0.243	0.539	0.335	0.167	0.653	0.276
SPPIDER	0.752	0.331	0.557	0.415	0.285	0.755	0.373
MaSIF-site	0.780	0.370	0.561	0.446	0.326	0.775	0.439
GraphPPIS	0.776	0.368	0.584	0.451	0.333	0.786	0.429
RGN	0.785	0.382	0.587	0.463	0.349	0.791	0.441
Seq-InSite	0.826	0.448	N/A	0.448	0.345	0.798	0.430
DeepProSite	0.842	0.501	0.443	0.470	0.379	0.813	0.490
AGAT-PPIS	0.856	0.539	0.603	0.569	0.484	0.867	0.574
GHGPR-PPIS	0.860	0.551	0.620	0.583	0.501	N/A	**0.596**
**MVSO-PPIS**	**0.868**	**0.574**	**0.630**	**0.601**	**0.523**	**0.873**	0.583

Bold values indicate the highest score in the corresponding evaluation-metric column.

Furthermore, comparative analyses were conducted between the model and AGAT-PPIS on three independent test sets: Test_315-28, Btest_31-6, and UBtest_31-6. As shown in Table 2, the results demonstrate that the model achieved significant improvements over AGAT-PPIS in terms of MCC and AUPRC metrics on the three independent test sets. By comparing the performance across the three independent test sets, the model is proven to possess stronger generalization capabilities and robustness compared to AGAT-PPIS.

**Table 2.** Performance comparison with AGAT-PPIS on Test_315-28 and UBtest31-6.

Method	Test_315-28		Btest31-6		UBtest31-6	
	MCC	AUPRC	MCC	AUPRC	MCC	AUPRC
AGAT-PPIS	0.481	0.572	0.485	0.583	0.327	0.365
GHGPR-PPIS	0.486	0.567	N/A	N/A	0.356	0.367
**MVSO-PPIS**	**0.510**	**0.583**	**0.544**	**0.593**	**0.357**	**0.397**

Bold values indicate the highest score in the corresponding evaluation-metric column.

### 3.2 Comparison of model performance: MVSO-PPIS with ProtT5 embeddings versus other models

Protein language model embeddings have shown great promise in protein binding site prediction by capturing rich contextual and semantic information from sequences. In this study, we adopt embeddings derived from the ProtT5-XL-UniRef50 model (ProtT5), a widely used protein language model based on the T5-3B architecture (Elnaggar *et al.* 2022). ProtT5 uses a Transformer-based architecture with a BERT-style masking strategy and is pretrained in a self-supervised manner on large-scale protein sequence data. The resulting residue-level embeddings (with a dimensionality of 1024) capture rich contextual and evolutionary information.

These embeddings are further utilized as node features in our graph-based MVSO-PPIS framework, enabling the integration of semantic sequence representations with spatial and structural cues for protein–protein interaction site prediction. The model is trained on the PP-1001_Train dataset and evaluated on the independent PP-250_Test set. To comprehensively and objectively assess the performance of MVSO-PPIS, we conducted a series of comparative experiments against several state-of-the-art protein–protein interaction (PPI) site predictors. As shown in Table 3, The experimental results indicate that MVSO-PPIS outperformed the GeoNet method by 0.1% in F1 score, 0.5% in Matthews Correlation Coefficient (MCC), and 1.1% in Area Under the Precision-Recall Curve (AUPRC). Experimental results demonstrate the superior predictive accuracy and generalization capability of our method with ProtT5 embeddings.

**Table 3.** Performance comparison with other models on P-250_Test.

Method	F1	MCC	AUPRC
SPPIDER	0.271	0.199	0.179
GraphPPIS	0.298	0.230	0.212
MaSIF-site	0.255	0.184	0.179
GeoNet	0.432	0.381	0.411
**MVSO-PPIS**	**0.433**	**0.386**	**0.422**

Bold values indicate the highest score in the corresponding evaluation-metric column.

### 3.3 Ablation experiments

#### 3.3.1 Model structure ablation experiments

In the model structure ablation experiments, two sets of experiments were conducted: one focusing on the ablation of the feature channels in MVSO-PPIS, and the other assessing the impact of using ATT Fusion. As shown in Table 3, available as supplementary data at *Bioinformatics* online, using both AGAT and SubGraph significantly enhances model performance compared to using only AGAT, and it also shows a marked improvement over using only SubGraph. As mentioned earlier, AGAT simulates the complete graph view of the protein, while SubGraph simulates the subgraph view.

**Figure 3.**
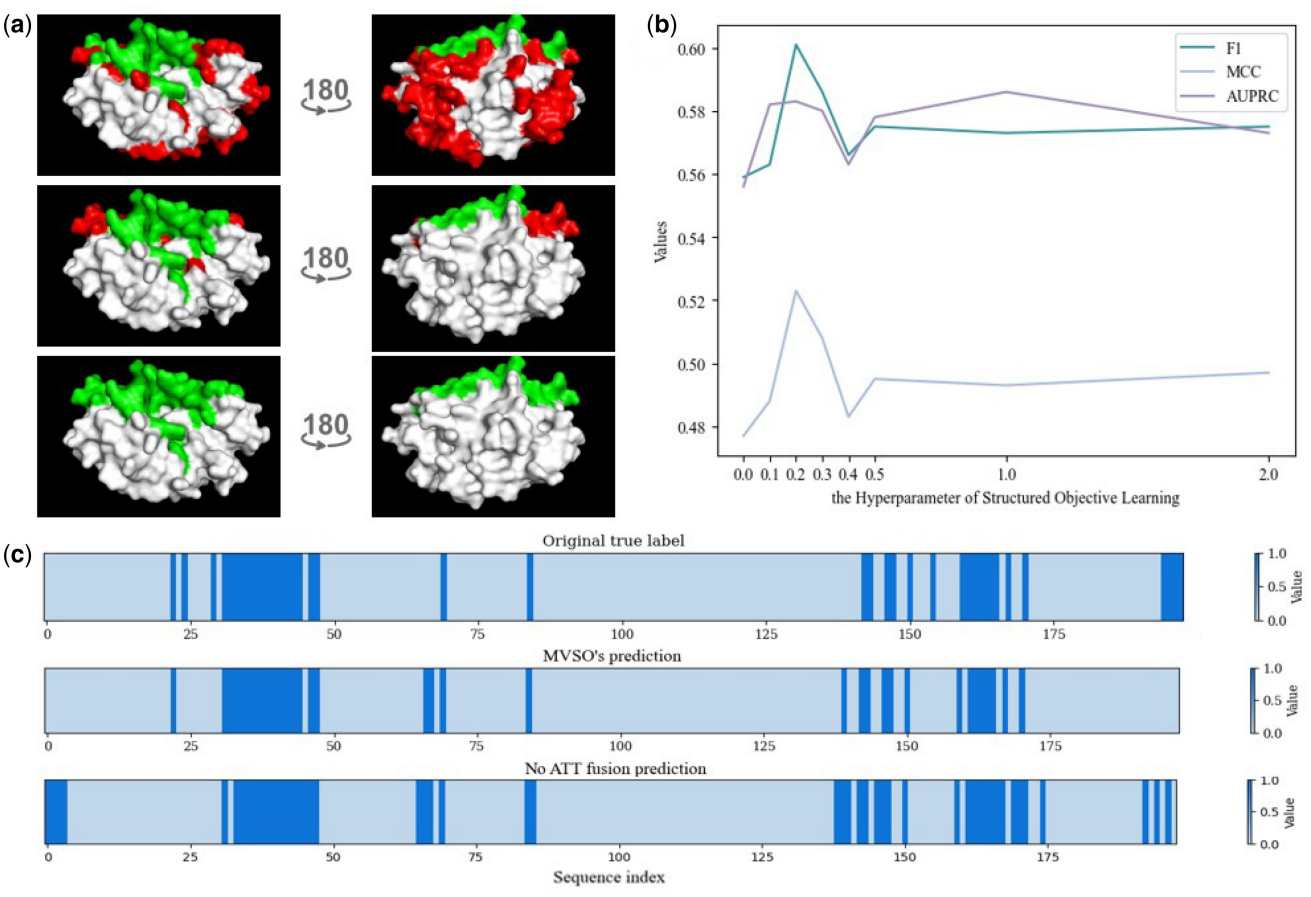
(a) The results for protein “3shgA” (PDB ID) demonstrate 3D structural models comparing AGAT-PPIS (top), MVSO-PPIS (middle), and ground truth labels (bottom), where green regions indicate correctly predicted residues while red regions denote prediction errors. (b) The Hyperparameter ablation experiment of Structured Objective Learning on Test_60. (c) Comparative performance analysis of MVSO-PPIS models with different feature fusion strategies on protein “3shgA” (PDB ID. The maximum sequence identity with the training set is 15.3%) at the sequence level. The top panel displays the ground truth distribution, the middle panel presents the prediction results from MVSO-PPIS incorporating ATT Fusion technology, while the bottom panel shows the baseline model predictions without this technique. In the visualization, darker regions represent predicted positive samples (protein binding sites), whereas lighter areas indicate predicted negative samples (non-binding site residues).

Furthermore, as depicted in Fig. 2, available as supplementary data at *Bioinformatics* online, we assessed the impact of using ATT Fusion on metrics such as F1, MCC, AUROC, and AUPRC on the independent dataset Test_60. It is evident that incorporating ATT Fusion yields the best results for the model that performs multi-view feature fusion. To further validate the effectiveness of this technique, we conducted a comparative analysis on protein “3shgA” (PDB ID) from the sequence view between the MVSO-PPIS model with ATT Fusion and its counterpart without this technology. As shown in Fig. 3c, the ATT Fusion-enhanced MVSO-PPIS model demonstrates significantly higher consistency with the ground truth labels in sequence feature representation. These results collectively affirm that using ATT Fusion techniques can effectively enhance the overall performance of the model. More details on the ablation studies can be found in the Supplementary Material.

#### 3.3.2 Assessing the effectiveness of structured objective learning

To assess SOL’s effectiveness, experiments compared its implementation in MVSO-PPIS and AGAT-PPIS. As shown in Table 4, AGAT-PPIS with SOL outperformed its non-SOL counterpart in all metrics except Recall. MVSO-PPIS with SOL showed notable improvements: ACC (+0.8%), Precision (+1%), Recall (+7.7%), F1 (+4.2%), MCC (+4.6%), AUROC (+2.8%), and AUPRC (+2.7%). Moreover, SOL-enhanced MVSO-PPIS achieved the highest scores in all metrics except AUPRC.

**Table 4.** The Effectiveness of structured objective learning comparison with AGAT-PPIS and MVSO-PPIS on Test_60.

Method	Structured objective learning	ACC	Precision	Recall	F1	MCC	AUROC	AUPRC
AGAT-PPIS	×	0.865	0.539	0.603	0.569	0.484	0.867	0.574
	✓	0.863	0.563	0.582	0.573	0.491	0.867	**0.602**
MVSO-PPIS	×	0.862	0.564	0.553	0.559	0.477	0.845	0.556
	✓	**0.868**	**0.574**	**0.630**	**0.601**	**0.523**	**0.873**	0.583

Bold values indicate the highest score in the corresponding evaluation-metric column.

To validate SOL’s structural enhancement, we analyzed the accuracy of predicted singular (“010”, “101”) and edge (“01”, “10”) structures for MVSO-PPIS, MVSO-PPIS (w/o SOL), and AGAT-PPIS (w/o SOL) on Test_60 and Test_315-28 (see Table 5). On Test_60, MVSO-PPIS achieved the highest accuracy for edge and “101” structures, though “010” accuracy was marginally lower (0.3%) than MVSO-PPIS (w/o SOL), likely due to Test_60’s limited data. On Test_315-28, MVSO-PPIS surpassed other methods in predicting both singular and edge structures. Comparisons across datasets confirmed SOL’s significant accuracy improvements for these structural predictions.

**Table 5.** Accuracy of target structure on Test_60 and Test315_28.

Method	DataSet	Sequence structure			
		01	10	010	101
AGAT-PPIS(without SOL)	Test60 (ACC)	0.313	0.314	0.194	0.246
MVSO-PPIS(without SOL)		0.315	0.335	**0.259**	0.228
**MVSO-PPIS**		**0.368**	**0.370**	0.256	**0.316**
AGAT-PPIS(without SOL)	Test315_28 (ACC)	0.249	0.297	0.210	0.218
MVSO-PPIS(without SOL)		0.334	0.339	0.267	0.243
**MVSO-PPIS**		**0.403**	**0.409**	**0.321**	**0.323**

Bold values denote the highest accuracy achieved in the respective column.

Figure 1, available as supplementary data at *Bioinformatics* online compares interaction site predictions for protein 1q9a chain A (Test_60) using MVSO-PPIS, MVSO-PPIS (without SOL), and ground truth labels. Green/red indicate correct/incorrect predictions. MVSO-PPIS preserved structural integrity in clustered sites (lower left), while MVSO-PPIS (without SOL) failed, demonstrating SOL’s efficacy for PPIS prediction.

For spatial compactness validation, we compared AGT-PPIS and MVSO-PPIS using protein 3shgA (Fig. 3a). Results show MVSO-PPIS predictions (middle) exhibit superior spatial compactness versus AGAT-PPIS (top), better matching ground truth (bottom) in spatial distribution. Mispredicted residues (red) are more clustered, confirming our model’s ability to maintain binding site structural integrity.

Finally, we executed a hyperparameter ablation experiment for SOL on Test_60. As shown in Fig. 3b, the hyperparameter of SOL reached maximum values for F1, MCC, and AUPRC when set to 0.2. This indicates that the model effectively learns the structural properties of interaction sites while ensuring the accuracy of predicted protein interaction sites. Therefore, we set the hyperparameter of SOL to 0.2.

## 4 Conclusion

In this study, we proposed MVSO-PPIS, a novel multi-view protein-protein interaction site prediction model combining a multi-view attention fusion architecture with Structured Objective Learning (SOL). The model extracts comprehensive protein features from complete-graph and subgraph views, fused via attention mechanisms, and optimizes binding site identification through SOL. It achieved superior performance on Test_315-28, Btest_31-6, and UBtest_31-6 datasets compared to baseline models.

Despite these promising results, two limitations should be noted. First, the generalizability of the model to alternative functional sites, such as small-molecule, catalytic, or metal-ion binding sites, and to diverse classes of protein-protein interfaces (e.g. enzyme-inhibitor, antibody-antigen, or receptor-ligand complexes) remains unverified. Second, inherent structural dependency, although partially alleviated by predicted conformations, can still compromise performance for orphan proteins, dynamic conformational ensembles, or distinct topological regions of protein surfaces (including grooves, protrusions, flat patches, or pocket rims). These limitations underscore critical avenues for future investigation.

## Supplementary Material

btaf470_Supplementary_Data

## Data Availability

The source code is available at https://github.com/Edwardblue282/ MVSO-PPIS(https://doi.org/10.6084/m9.figshare.29580308.v1).
