# Supplementary material for: MVSO-PPIS: a structured objective learning model for protein–protein interaction sites prediction via multi-view graph information integration
Source: Bioinformatics. 2025 Sep 1;41(9):btaf470. doi: 10.1093/bioinformatics/btaf470 (PMC12462378; doi:10.1093/bioinformatics/btaf470)
Supplement: btaf470_Supplementary_Data [file btaf470_supplementary_data.docx]

**Protein representation**

Once the adjacency matrix A is generated, the original edge feature vector set is represented as $E^{\mathrm{raw}}=\left\{ e_{\mathrm{ij}}^{\mathrm{raw}} | A_{\mathrm{ij}}=1 \right\}$, where $e_{\mathrm{ij}}^{\mathrm{raw}}$ is the original edge feature vector between nodes i and j. The feature vector encompasses two attributes: (i) the Euclidean distance $D_{\mathrm{ij}}$ between nodes i and j; and (ii) the cosine of the angle $\cos(\theta_{\mathrm{ij}})$ between nodes i and j relative to the reference node o, calculated as shown in Equation S1. The original edge feature $e_{\mathrm{ij}}^{\mathrm{raw}}$ is normalized to a range of 0 to 1, endowing the model with translational and rotational invariance.

$\cos(\theta_{\mathrm{ij}})=\frac{\vec{P_{0}P_{i}}\vec{P_{0}P_{j}}}{\left| \vec{P_{0}P_{i}} \right|\left| \vec{P_{0}P_{j}} \right|}$ (S1)

**Protein graph subgraph construction**

Identifying the crucial substructures within proteins is of paramount importance, given that protein binding sites constitute a subset of these structural elements.Based on the Graph U-Net method [1], a graph pooling module (Figure 1(C)) has been designed to obtain subgraphs, enabling subsequent graph convolutional networks to learn the importance of protein residues in contributing to protein binding sites. The graph pooling module leverages the node feature matrix of the original graph to partition subgraphs using low-dimensional mapping and importance-based ranking. Subsequently, it selects the subgraph's node feature matrix $X^{'}$ and adjacency matrix $A^{'}$ from the original protein graph's node feature matrix $X$ and adjacency matrix $A$ based on the indices $\mathrm{idx}$ obtained from the partitioning.

$y=\frac{\mathrm{Xp}}{\left\| p \right\|}$ (S2)

$\bar{y}=sigmoid(y)$ (S3)

$idx=rank(\bar{y}, k)$ (S4)

$\bar{X}=X(idx, :)$ (S5)

$A^{'}=A^{2}(idx, idx)$ (S6)

$X^{’}=\bar{X}\odot\bar{y}^{1_{C}^{T}}$ (S7)

Where $\mathrm{Xp}$ indicates that the node feature matrix X and the mapping vector p are multiplied.The operation $rank(\bar{y}, k)$ is designed to sort and select the $k$ largest values in $\bar{y}$, and return their indices ($\mathrm{idx}$).$X(idx, :)$ and $A^{2}(idx, idx)$ are extracted from the original feature matrix and adjacency matrix based on $\mathrm{idx}$, forming the node feature matrix $\bar{X}$ and adjacency matrix $A^{'}$ of the subgraph.$1_{C}^{T}$ denotes a vector of size $C$ with all components equal to 1.

The node feature matrix $X$ is multiplied by the mapping vector $p$ to obtain $y$, which is then processed by the sigmoid function to yield $\bar{y}$(Equation S2,3). Subsequently, $\bar{y}$ and k are used to perform a rank operation to obtain the indices $\mathrm{idx}$ of the retained features (Equation S4). The rank operation involves sorting $y$ based on score ranking, where $k$ represents the number of top-scoring features to be selected. Based on the derived idx, the node feature matrix $X$ and the adjacency matrix $A$ are filtered to extract features (Equations S5,S6), resulting in the subgraph's node feature matrix $X^{‘}$ and adjacency matrix $A^{'}$. The notation $1_{C}^{T}$ represents a vector of size $C$ with all components equal to 1. During the node selection process, the mapping vector $p$ is learnable, allowing the method to learn how to select important nodes within the subgraph. Additionally, to facilitate feature fusion with the node features learned from the complete graph, after updating the features on the subgraph, the subgraph is restored to the size of the original graph through an inverse operation (Equation S8).$0_{N\times C}$ represents a zero matrix of size $N\times C$.

$X^{''}=distribute(0_{N\times C}, X^{’}, idx)$ (S8)

**Experimental setup**

In this experiment, we employed 5-fold cross-validation on the training set to assess model performance. The average AUROC and AUPRC served as key metrics for evaluating the model, guiding the selection of suitable features and hyperparameters. Subsequently, the entire training set was utilized for the final model training. Drawing on prior experience and conducting necessary experiments, we determined and set the final hyperparameters accordingly.The cutoff distance for protein edges was set to 14 Å. The subgraph splitting ratio was set to 0.7, and the number of subgraph layers was set to 3. For the AGAT complete graph channel, the number of layers was set to 8. The output dimensions of the final three MLP layers were set to 256, 128, and 2, respectively. Adam optimizer were used to optimize the model. A learning rate of 0.001 was used during training for 100 epochs. All experiments were conducted on a workstation equipped with an NVIDIA RTX 4090 GPU (24 GB VRAM), enabling efficient training and inference of deep learning models.

**More details about Datasets**

The datasets used in this experiment were fine-tuned according to the datasets used by GraphPPIS [2], including the training set (Train_335) and test sets (Test_60, Test_315 and UBtest_31). Train_335 and Test_60 were derived from the widely used public datasets Dset_186, Dset_72 [3], and Dset_164 [4]. These datasets underwent a six-step filtering process [2] and used BLASTClust [5] to remove redundant protein sequences with high sequence similarity (sequence identity > 25%) or overlap (E-value < 1e-6) to ensure the high quality and low redundancy of the datasets. Test_315 is another test set constructed by Yuan Q et al. [2] for further verifying the generalization capability of the model. Ubtest_31 contains 31 unbound protein structures corresponding to 31 proteins in Test_60 that have known monomeric structures in PDB for evaluating the robustness of the model and the impact of the conformational changes on model performance. The specific statistical details of the datasets mentioned above are shown in Supplementary Table S1 and the bold fonts are the adjusted datasets used in our experiments. During the experiments, it was found that a few protein sequences in these datasets were not consistent with the sequences of those in the corresponding protein pdb files from PDB website. Therefore, these abnormal proteins were eliminated in this experiment. The details of the adjusted datasets are shown in bold fonts in Supplementary Table S1.

Table S1. The statistical details of training and test sets

| Dataset | Protein chains | Interacting residues | Non-interacting residues | of interacting residues (%) |
| --- | --- | --- | --- | --- |
| Train_335 | 335 | 10374 | 55992 | 15.63 |
| Train_335-1 | 334 | 10336 | 55872 | 15.61 |
| Test_60 | 60 | 2075 | 11069 | 15.79 |
| Test_315 | 315 | 9355 | 55976 | 14.32 |
| Test_315-28 | 287 | 8566 | 51810 | 14.19 |
| Ubtest_31 | 31 | 841 | 5813 | 12.64 |
| Ubtest_31-6 | 25 | 711 | 5206 | 12.02 |
| PP-250_Test | 250 | 6386 | 73391 | 8.0 |
| PP-1001_Train | 1001 | 25172 | 264564 | 8.7 |

**Subgraph division ratio ablation experiments**

To investigate the impact of subgraph perspective scaling on model performance, we conducted an ablation experiment on the Subgraph division ratio using the independent dataset Test_60. As shown in Supplementary Table S2, the model exhibited local maxima in F1 and MCC at Subgraph division ratios of 0.3 and 0.7, respectively, while local maxima in AUPRC were observed at ratios of 0.3 and 1.0. Ultimately, considering F1, MCC, and AUPRC collectively, the model performed optimally at a Subgraph division ratio of 0.7.

Table S2. the Hyperparameter ablation experiment of Subgraph division radio on Test_60

| Method | Subgraph division radio | ACC | Precision | Recall | F1 | MCC | AUROC | AUPRC |
| --- | --- | --- | --- | --- | --- | --- | --- | --- |
| MVSO-PPIS | 0.1 | **0.870** | **0.605** | 0.508 | 0.553 | 0.480 | 0.840 | 0.561 |
|  | 0.3 | 0.865 | 0.570 | 0.585 | 0.577 | 0.496 | 0.866 | **0.594** |
|  | 0.5 | 0.868 | 0.596 | 0.514 | 0.552 | 0.477 | 0.854 | 0.563 |
|  | **0.7** | 0.868 | 0.574 | **0.630** | **0.601** | **0.523** | **0.873** | 0.583 |
|  | 1.0 | 0.867 | 0.574 | 0.611 | 0.592 | 0.513 | 0.872 | 0.593 |

**Evaluation metrics**

Protein site prediction is approached as a binary classification challenge, focusing on identifying binding sites among amino acids on a protein by extracting features through a model-based framework. For the rigorous evaluation of the model's performance, a set of standard binary classification metrics has been employed. These metrics encompass Accuracy, calculated as the ratio of correct classifications to the total number of instances processed; Precision, which is the proportion of true positive predictions among all positive predictions; Recall, representing the fraction of true positive predictions relative to all actual positives; F1 Score, a harmonic mean of Precision and Recall that balances these two metrics; Matthews Correlation Coefficient (MCC), quantifying the correlation between actual and predicted classifications; Area Under the Receiver Operating Characteristic Curve (AUROC), assessing the model's ability to differentiate between classes; and Area Under the Precision-Recall Curve (AUPRC), addressing class imbalance and evaluating performance across different thresholds. These metrics are derived from the fundamental counts of true positives (TP), true negatives (TN), false positives (FP), and false negatives (FN), as detailed in Equation 22-26.

$Accuracy=\frac{TP+TN}{TP+TN+FP+FN}$ (1)

$Precision=\frac{TP}{TP+FP}$ (2)

$Recall=\frac{TP}{TP+FN}$ (3)

$F1=\frac{2*Precision*Recall}{Precision+Recall}$ (4)

$MCC=\frac{TP*TN-FP*FN}{\sqrt{\left( TP+FP \right)*\left( TP+FN \right)*\left( TN+FP \right)*\left( TN+FN \right)}}$ (5)

**Feature ablation experiments**

Following the framework of AGAT-PPIS, the importance of PSSM, HMM, and DSSP matrices has been validated in previous studies; thus, we did not conduct further verification here. Instead, we demonstrated the significance of using different combinations of node features. As shown in Table S2, when the model input excludes the Pseudo-position Embedding, there is a slight improvement in ACC and Precision, but notable declines are observed in Recall, F1, MCC, AUROC, and AUPRC. This indicates that relying solely on Atomic Features leads the model to favor predicting the more prevalent class, resulting in an inadequate understanding of the imbalanced data distribution of protein binding sites. Conversely, when the model input excludes Atomic Features, all performance metrics decline. This demonstrates that atomic features play a crucial role in enhancing model performance. Therefore, every feature in the selected combination of node features is essential for the model.

Table S2.Performance comparison of MVSO-PPIS with different feature groups on independent test set Test_60

| Method | Feature | ACC | Precision | Recall | F1 | MCC | AUROC | AUPRC |
| --- | --- | --- | --- | --- | --- | --- | --- | --- |
| MVSO-PPIS | HMM+PSSM+DSSP | 0.820 | 0.453 | 0.558 | 0.489 | 0.345 | 0.798 | 0.485 |
|  | HMM+PSSM+DSSP+Atomic Features | **0.869** | **0.588** | 0.559 | 0.573 | 0.496 | 0.858 | 0.578 |
|  | HMM+PSSM+DSSP+Pseudo-position Embedding | 0.839 | 0.492 | 0.528 | 0.509 | 0.414 | 0.819 | 0.495 |
|  | **All**  **(HMM+PSSM+DSSP+Pseudo-position Embedding**  **+Atomic Features)** | 0.868 | 0.574 | **0.630** | **0.601** | **0.523** | **0.873** | **0.583** |

Table S3. Performance comparison of MVSO-PPIS with different feature channel on independent test set Test_60

| Method | Feature channel | ACC | Precision | Recall | F1 | MCC | AUROC | AUPRC |
| --- | --- | --- | --- | --- | --- | --- | --- | --- |
| MVSO-PPIS | AGAT | 0.859 | 0.547 | 0.607 | 0.576 | 0.492 | 0.859 | 0.572 |
|  | SubGraph | 0.738 | 0.266 | 0.375 | 0.311 | 0.158 | 0.637 | 0.256 |
|  | **AGAT+SubGraph** | **0.868** | **0.574** | **0.630** | **0.601** | **0.523** | **0.873** | **0.583** |


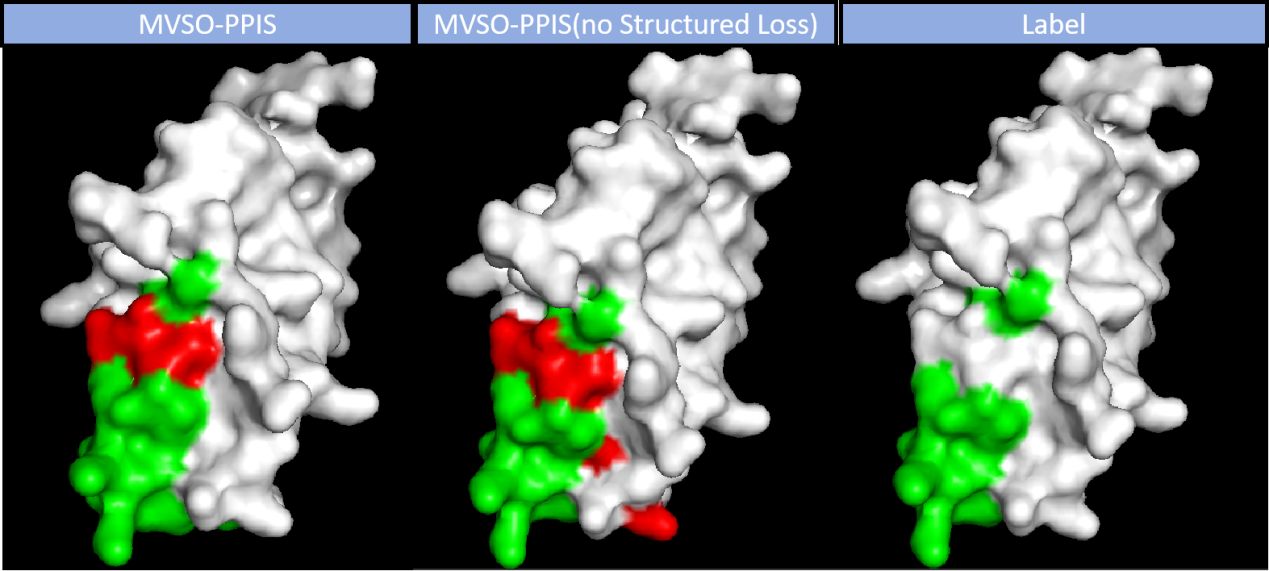


Figure S1. the prediction results for the interaction sites of protein 1q9a (PDB ID) chain A from Test_60, comparing MVSO-PPIS, MVSO-PPIS (without SOL), and the actual labels


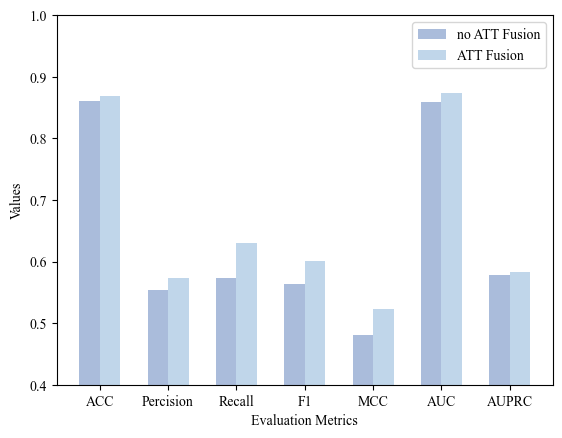


Figure S2. Performance comparison of MVSO-PPIS with different feature fusion strategy on independent test set Test_60

**References**

1. H. Gao and S. Ji. Graph u-nets. In international conference on machine learning, pages 2083–2092. PMLR, 2019.
2. Yuan Q, Chen J, Zhao H, et al. Structure-aware protein–protein interaction site prediction using deep graph convolutional network. Bioinformatics 2022; 38: 125-132.
3. Murakami Y, Mizuguchi K. Applying the Naïve Bayes classifier with kernel density estimation to the prediction of protein–protein interaction sites. Bioinformatics 2010; 26: 1841–1848.
4. Dhole K, Singh G, Pai P P, et al. Sequence-based prediction of protein–protein interaction sites with L1-logreg classifier. J THEOR BIOL 2014; 348: 47–54.
5. Altschul S F, Gish W, Miller W, et al. Basic local alignment search tool. J MOL BIOL 1990; 215: 403–410.
